# Exemplar-based image completion using image depth information

**DOI:** 10.1371/journal.pone.0200404

**Published:** 2018-09-13

**Authors:** Mang Xiao, Guangyao Li, Li Xie, Lei Peng, Qiaochuan Chen

**Affiliations:** 1 School of Computer Science and Information Engineering, Shanghai Institute of Technology, Shanghai, China; 2 College of Electronics and Information Engineering, Tongji University, Shanghai, China; 3 School of Information Engineering, Tai’an college, Shandong, China; Yonsei University, KOREA, REPUBLIC OF

## Abstract

Image completion techniques are required to complete missing regions in digital images. A key challenge for image completion is keeping consistency of image structures without ambiguity and visual artifacts. We propose a novel method for image completion using image depth cue. Our method includes three major features. First, we compute the image gradient to improve image completion when searching for the most similar patches. Second, using image depth, we guide image completion by means of appropriate scale transformation. Third, we propose a global optimization patch-based method having gradient and depth features for image completion. Experiments demonstrate that our approach is a potentially superior method for completing missing regions.

## Introduction

Image completion techniques are used to complete target regions (i.e., “holes”) in digital images. From a computational perspective, this is a difficult problem, because the completed image must be credible and consist of realistic shapes and textures. Existing image completion techniques can be roughly divided into two main categories [1]: diffusion-based and exemplar-based (or patch-based).

Diffusion-based [2, 3] methods complete missing image regions by using thermal diffusion equations that propagate image information from surrounding regions into damaged regions. These methods include Euler’s elastic model [4] and the total variation models [5]. They perform effectively in small target areas. However, they are prone to blurring when the damaged region is large.

Exemplar-based [6] methods have been proposed for completing large damaged regions. These methods [7] process images in a greedy manner, which results in visually implausible regions. Many globally optimized approaches [8], [9], [10], [11] have been proposed to address this problem. Wexler et al. [8] employed an optimization method that employs a well-defined objective function that constricts the global visual coherence. This method is computationally expensive. However, a fast PatchMatch method [12] considerably reduces running time. Because patch translation is difficult to obtain for the full structure of an image, some methods [13], [14], [15], [16] have adopted both photometric and geometric transformational processes to address this issue.

**Fig 1:**
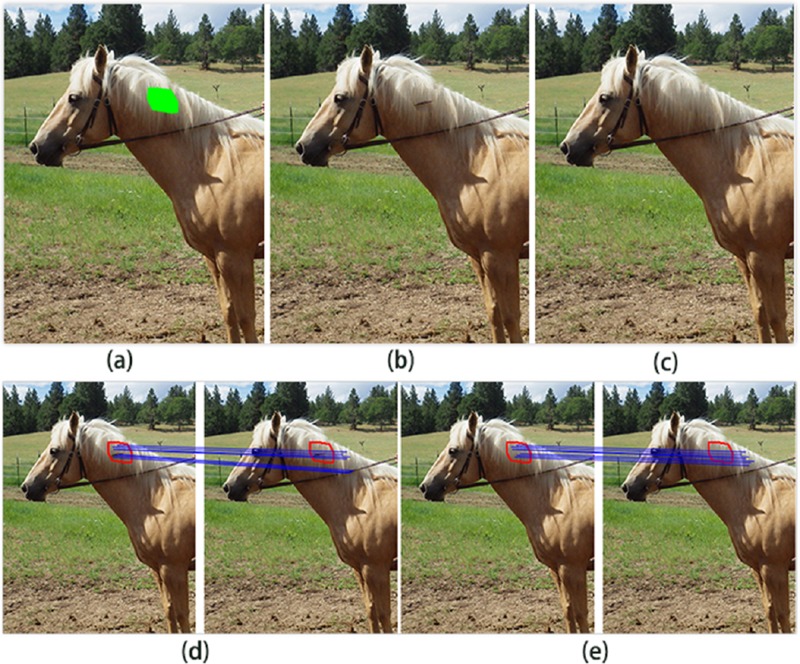
Gradient features for image completion: (a) input image, (b) result of Barnes et al [12], (c) result of our method, (d) approximate nearest neighbors without gradient features, (e) approximate nearest neighbors with gradient features.

However, these methods can easily produce mistakes. For example, as shown in Fig 1(a), the source patches in the red rectangle are directly used to fill the target region in blue rectangle. That yield in visual implausible results, especially, visual artifacts such as a more distant lantern being bigger than a closer lantern, as shown in Fig 1(b). The main reason for these production errors is that the algorithms are mostly incapable of selecting “correct” patches and not transforming the source patches for target patches. The depth-aided exemplar-based method proposed by Xu et al. [17] uses information taken from a depth image to attain higher visual quality than previous approaches. The depth of an image is so critical to image analysis and understanding that many studies [18, 19] have examined it for many years. Saxena et al. [19] adopted a Markov random field (MRF) to predict the depth of an image and developed qualitatively correct three dimensional (3D) models.

The following describes the three main features of the proposed method in detail. First, we compute the image gradient to improve image completion when searching for the most similar patches. Second, using image depth, we guide image completion by means of appropriate scale transformation. Third, we propose a global optimization patch-based method having gradient and depth features for image completion.

## Exemplar-based image completion using image depth information

We divide the images into a target region T and source regions S. The target region in the damaged image may be inconsistent because of inaccurate patches, geometric transformations, or variable spatial illumination. To address the optimization problem of filling the target regions, we use the new energy function in the following equation.

$$\begin{array}{c} \begin{array}{cc} E(T,S)= & \sum_{q\subset T}\min_{p\subset S}(\lambda_{1}E\left(Q,P\right)+\lambda_{2}E\left(\nabla Q,\nabla P\right)\\ & +\lambda_{3}E\left(DQ,DP\right)) \end{array} \end{array}$$ (1)

where *Q* = *N*(*q*) denotes a target patch of size *w* × *w*, which appears near pixel *q* at the patch’s top left corner, *P* = *f*(*N*(*p*)) denotes a *w* × *w* patch, which is a source patch result from a consequence of a small neighborhood N around pixel p and undergoes photometric and geometric transformation. We define each patch as having five channels at every pixel (*L*, *a*, *b*, ∇_*x*_ *L*, ∇_*y*_ *L*), in (L*, a*, b*) color space, (*L*, *a*, *b*) denotes three color channels and denotes two gradient channels to estimate the change of the luminance. For simple symbols, we define Q (or P) as the patch’s three color channels, ∇*Q*, ∇*P* as the luminance’s two gradient channels, and *DQ*, *DP* as the depth of the target and source patches. λ_1_, λ_2_, λ_3_ are the weights of three terms, whereas *f* denotes the transformation, which includes the rotation, translation, reflection, and non-uniform scale.

### Color cost

We define the color term using the following function:

$$\begin{array}{c} E\left(Q,P\right)={∥Q_{i}-P_{i}∥}^{2} \end{array}$$ (2)

where *Q*_*i*_ is the color of the i-th pixel in the target patch Q and *P*_*i*_ is the color of the i-th pixel in the source patch *P*. Using the *l*^2^ norm, we then measure the similarity between patches.

### Gradient cost

**Fig 2:**
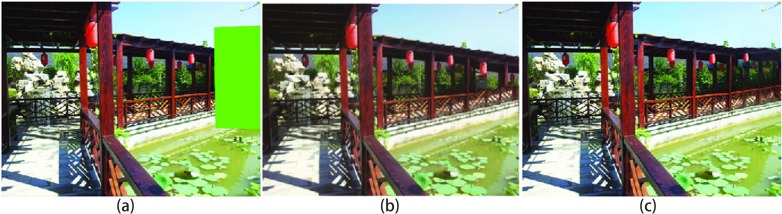
Depth for image completion: (a) input image, (b) results of the Barnes method [12], (c) results of our method.

To improve the results of patch-based inpainting method, obtaining “correct”patches is necessary. However, two main factors result in incorrect patches. The first is the use of only *l*^2^ patch distance to compute the similarity between patches. The second is that PatchMatch [12] itself may recognize patches incorrectly. The problem is illustrated in Fig 2. The approach of Barnes [12] could not locate the correct texture because it does not possess a gradient feature. As shown in Fig 2(d), for the rein to have patches of similar color to that of the mane, the Barnes approach mistakes the mane for the correct region. Adopting the gradient feature, we identify the correct region for the target region, as shown in Fig 2(e).

The gradient feature term is defined as follows:

$$\begin{array}{c} E\left(\nabla Q,\nabla P\right)={∥{\nabla Q}_{i}-{\nabla P}_{i}∥}^{2} \end{array}$$ (3)

Where ∇*Q_i_* is the gradient of the i-th pixel in the target patch *Q* and ∇*P_i_* is the gradient of the i-th pixel in the source patch *P*. We also adopt *l*^2^ norm to measure the gradient similarity between patches.

### Depth-guided cost

**Fig 3:**
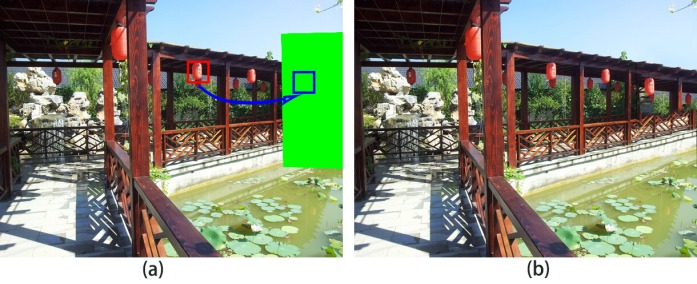
No transformation for image completion: (a) no transformational source patch, (b) results of the Barnes method [12].

To ensure the image completion result corresponds to visual semantics in the real world, it is critical to obtain the image’s depth information. In general, most images display the depth information related to objects in a scene except in the simple case of a facade. However, based on the visual semantics of the human eyes, the similar object that has different deep information should have a different size. For example, the size of an object of shallow depth is bigger than that of an object of deep depth, such as the lanterns shown in Fig 3(a).

However, most previous methods [8, 12, 20] assume that all objects have the same depth in a scene. These methods produce artificial results having wrong visual semantics. As shown in Fig 3(b), the lantern having deep depth is bigger than the near lantern having shallow depth.

**Fig 4:**
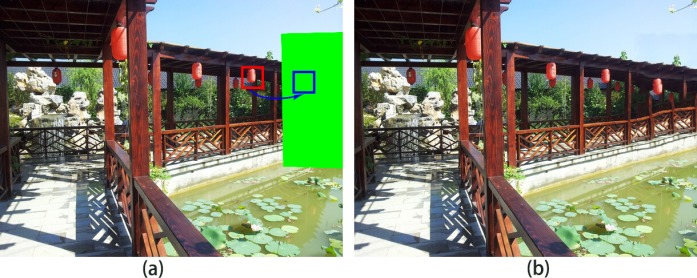
Inappropriate transformation for image completion: (a) inappropriate transformational source patch, (b) results of the Darabi method [20].

The primary reason for these production errors is that the algorithms do not know the scale relation between source patch and target patch, such as enlarged or reduced. The algorithm of Barnes et al. [12] is mostly incapable of selecting “correct” patches and does not transform the source patches into target patches. As shown in Fig 1(a), the source patches in the red rectangle are directly used to fill the target region in blue rectangle. The unsatisfactory results are shown in Fig 1(b), where the completed lantern is bigger than a closer lantern, and the railings have structural discontinuity. The method of Darabi et al. [20] utilized inappropriate transformation of source patches for target patches. As shown in Fig 4(a), the inappropriate transformed source patches in the red rectangle are used to fill the target region. The artificial results are shown in Fig 4(b); it can be inferred that due to the enlarged scale source patches, the completed lantern is bigger than a closer lantern and the railings are also bigger than closer railings, which results in wrong visual semantics.

Therefore, the critical factors of image completion include the selection of the appropriate source patch region and appropriate transformation. Johannes Kopf et al. [21] has confirmed that if the structure of close known region and farther known region are similar with the structure of target region, then the close known region can be a more effective improvement for the quality of image completion. In general, the depth difference is little between the close known region and target region. Therefore, the patches from the close known region are used to fill the target region to the maximum possible extent. On the other hand, using the depth of image, we can estimate the rough scale relation between source patch and target patch. Therefore, through appropriate transformation of source patches and optimization, the results of image completion can be improved.

**Fig 5:**
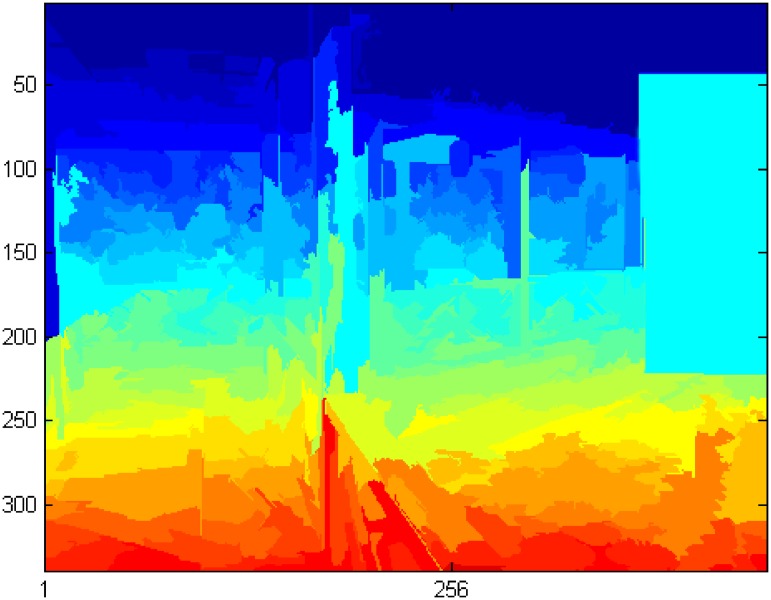
Image depth.

We obtain the depth of an image using the method of Saxena et al. [19], which uses a Markov Random Field to predict depth of image, as shown in Fig 5.

**Fig 6:**
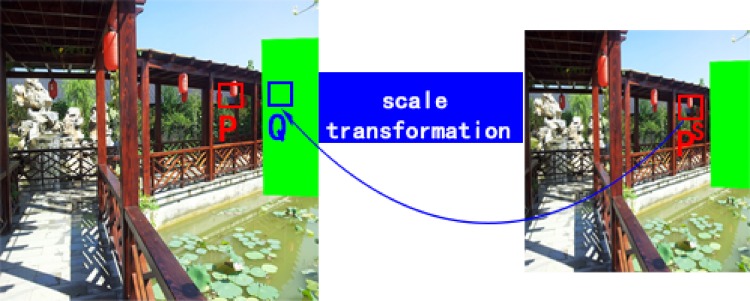
Scale transformtion for image completion.

The depth-guided term is defined as

$$\begin{array}{c} \begin{array}{cc} E(DQ,DP)= & W_{depth}(Q_{i}){∥{DQ}_{i}-{DP}_{i}∥}^{2}\\ & +W_{scale}(Q_{i})|\alpha \frac{DP_{i}}{DQ_{i}}-P_{i}^{s}| \end{array} \end{array}$$ (4)

where *DQ*_*i*_ is the depth of the i-th pixel in the target patch Q and *DP*_*i*_ is the depth of the i-th pixel in source patch *P*. Using the *l*^2^ norm, we then measure the depth between patches. The depth term encourages the target patch to choose the source patch that has a similar depth. We define $W_{depth}=W_{scale}=2^{-d\left(Q_{i}\right)}$, where *d*(*Q*_*i*_) is the distance of pixel *Q*_*i*_ to the nearest known pixel. $P_{i}^{s}$ is the current scale of pixel *P*_*i*_, as shown in Fig 6. In general, visually speaking, the scale and depth are linearly related [22], $\frac{scale(Q)}{scale(P)}=\alpha \frac{DP}{DQ}$. We use scaling factor *α* to adjust the scale transformation. Therefore, using this term, the inappropriate scale patches are penalized. The results of Fig 3(a) when using our method are shown in Fig 3(c).

### Patch searching and pixel filling

In general, given the large solution space and cost of evaluating the energy in a single solution, obtaining a globally optimal completion of the image is difficult. The method proposed by Wexler et al. [8] is an approximate optimization scheme that comprises two iterative steps called patch searching and pixel filling. Our algorithm is optimized based on this model.

#### Patch searching

For every target patch in the damaged image, the nearest neighbor patch must be found in the known region in order to minimize the value of Eq 1. We extend the PatchMatch algorithm that not only handles translations, scales, and rotations, but also copes with non-uniform scale and reflections.

To obtain invariance for small illumination, color changes, and exposure, we follow HaCohen et al. [23] and adopt bias b and gain g adjustments in three channels of a source patch. This allows the source patch to obtain the best matching target patch. We define bias and gain as:

$$\begin{array}{c} b(P^{c})=min\;\{max\;\{\mu (Q^{c})-g(P^{c})\;\mu \;(P^{c}),b_{min}\},b_{max}\} \end{array}$$ (5)

$$\begin{array}{c} g(P^{c})=min\{max\{\frac{\sigma (Q^{c})}{\sigma (P^{c})},g_{min}\},g_{max}\} \end{array}$$ (6)

where *c* denotes the color in each channel (*L*, *a*, *b*), *σ*() and *μ*() are the standard deviation and mean of the patch at each channel *c*, [*b*_*min*_, *b*_*max*_] and [*g*_*min*_, *g*_*max*_] are the bias and gain ranges, respectively, which are applied to regulate the colors of the patch *P*^*c*^: *P*^*c*^ ← *g*(*P*^*c*^)(*P*^*c*^) + *b*(*P*^*c*^).

#### Pixel filling

Eq 1 refers to all patch terms. Thus, the optimal damaged image satisfies the following function:

$$\begin{array}{c} T=\underset{I}{argmin}\{\lambda_{1}E\left(I,\bar{T}\right)+\lambda_{2}E\left(▽I,\bar{▽T}\right)+\lambda_{3}E\left(DI,\bar{DT}\right)\} \end{array}$$ (7)

Where $\bar{T}$ and $\bar{▽T}$ are images that have the same size as *I*. The value of pixel (i,j) in $\bar{T}$, $\bar{▽T}$ or $\bar{DT}$ is computed as follows:

T¯(i,j)=1w2∑k=0...w-1l=0...w-1NN(Qi-k,j-l)(k,l), (8)

▽T¯(i,j)=1w2∑k=0...w-1l=0...w-1▽NN(Qi-k,j-l)(k,l) (9)

DT¯(i,j)=1w2∑k=0...w-1l=0...w-1DNN(Qi-k,j-l)(k,l) (10)

Where *NN*(*Q*_*i*,*j*_) denotes the nearest neighbor source patch to target patch *Q*_*i*,*j*_, and the selected pixel (*k*, *l*) is defined as *NN*(*Q*_*i*,*j*_)(*k*, *l*) in that patch. $\bar{T}$ denotes the average colors of the target region that is filled with the overlapping transformed patches. $\bar{▽T}$ and $\bar{DT}$ are computed in the same manner.

## Results

Experiments for this study were performed using a computer with an Intel *Core*_*TM*_ i7-4700k 3.5 GHz processor. We set the patch size to 7 × 7. We defined the search range as [0.8, 1.3] for a uniform scale, [0.9, 1.1] for horizontal or vertical scales, and [−*π*/2, *π*/2] for rotation. The range of the bias for all three channels was [−10, 10] and for the gain was [0.8, 1.3]. We set the color weight to λ_1_ = 0.5, the gradient weight to λ_2_ = 0.2 and the depth weight to λ_3_ = 0.3. Scaling factor *α* is limited to the range [0.1, 1]. In our experiment, the scale factor is was set to 0.3. The PatchMatch iteration range was set to [20, 30] in order to update the nearest neighbor field. The algorithm was fairly robust with these parameters.

We compared our image completion approach with methods of Darabi et al. [20], Barnes et al. [12] and Huang et al. [16] to demonstrate the efficiency and robustness of the proposed algorithm.

**Fig 7:**
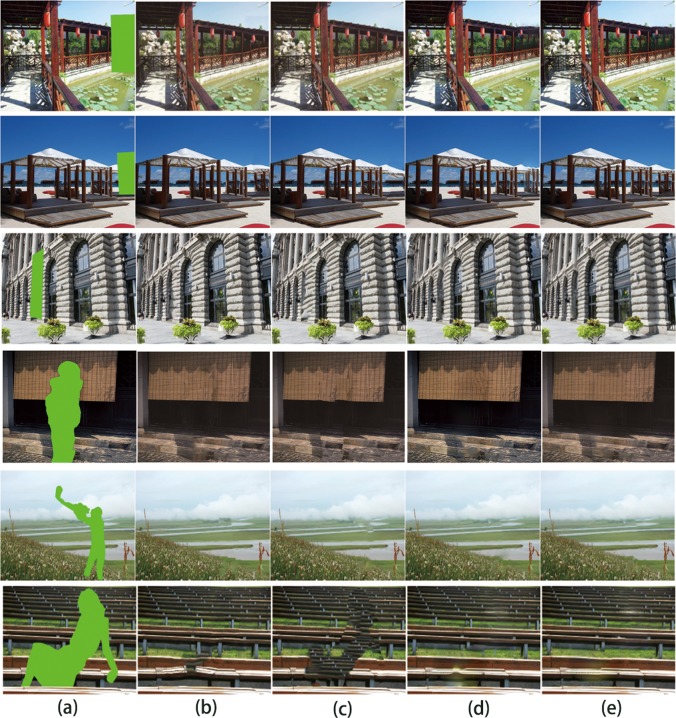
Comparison of relevant results: (a) damaged images, (b) Darabi’s results [20], (c) Barnes’ results [12], (d) Huang’ results [16], (e) our results.

The approaches proposed by Darabi et al. [20] and Barnes et al. [12] are adequate to fill the missing regions in a simple scene, that is, one in which images appear in the same planar or have the same depth. However, most scenes include many objects having different depth, such as the lantern, pavilion, building, stair, and lake in Fig 7.

### Image completion performance measured in human visual system

Fig 7 shows the results obtained using the three approaches on six damaged images. Results from the Darabi method, as shown in Fig 7, show considerable inconsistencies in Rows 1, 2, 4, and 6, which are easily produced by using only a color term to search for the best similar patch. Results from the Barnes method, as shown in Fig 7, show poor performance and artifacts for Rows 1, 2, 3, 4, and 6. This is the main reason that using PatchMatch to find appropriate patches for image completion without depth information is difficult. Results from the Huang method, as shown in Fig 7, show poor performance and artifacts for Rows 2, 4, 5 and 6. This is the main reason that using vanish point to obtain the planar information is difficult for these scenes. In addition, our approach obtained satisfactory results using depth information and gradient features. Therefore, our method performed better than existing methods in terms of both continuity and the visual effects.

### Image completion performance measured in PSNR

To find a satisfactory completion for the user is the real purpose of image completion. One important test is visual inspection and another one is obtaining quantitative results using Peak Signal to Noise Ratio (PSNR) [24]. The PSNR comparison of six images in Fig 7 is shown in Table 1.

**Table 1:** Image completion performance measured in PSNR.

Examples	Our	Darabi [20]	Barnes [12]	Huang [16]
(1)	23.56	22.67	23.32	23.21
(2)	22.22	21.14	21.76	21.62
(3)	23.18	22.69	22.41	22.98
(4)	25.22	23.68	24.45	24.73
(5)	22.73	21.86	22.38	22.19
(6)	24.92	23.25	24.47	24.26
mean	23.64	22.55	23.13	23.17

**Fig 8:**
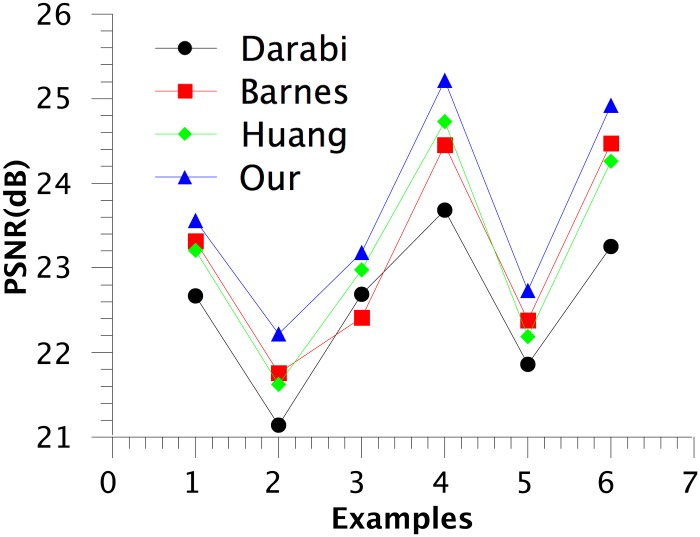
PSNR comparison.

The objective of image completion is to complete an image in the most satisfactory manner. Some primary methods [24], [25], [26] exist to measure the quality of the result. The first is visual inspection; the second is peak signal to noise ratio (PSNR). As shown in Fig 8, we compared the PSNR of six images. The results of Barnes show a gain of 0.58 dB in mean performance over the baseline, the results of Huang show a gain of 0.62 dB in mean performance over the baseline, whereas the mean performance of our method is 1.09 dB over the baseline. The PSNR comparison reveal that our approach performs more effectively than the other two methods.

### Image completion performance measured in SSIM

As the third method of image quality assessment, structural similarity (SSIM) [26] is adopted to assess the quality of image completion.

**Fig 9:**
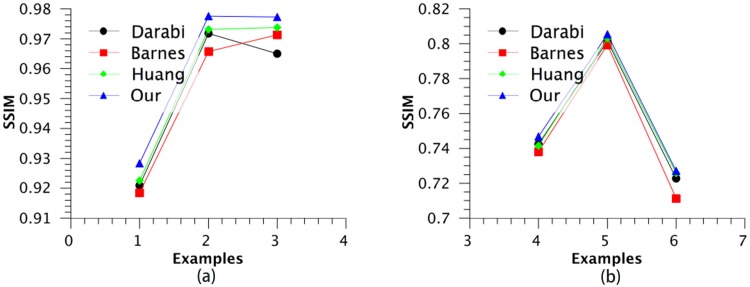
SSIM comprarison: (a) the results of the first three images in Fig 7 measured in SSIM, (b) the results of the last three images in Fig 7 measured in SSIM.

There are two different categories of images for image completion, as shown in Fig 7. The first three images in Fig 7 from the first category, where the removed content of target region is the real part of the scene. The last three images in Fig 7 are from second category, where the removed content of target region is not the real part of the scene but is another occlusion, such as a person. In general, the occlusion differ with the content of scene image. The SSIM value of the second category is significantly smaller than the SSIM value of the first category. For the first category, as shown in Table 2 and Fig 9(a), the results of Darabi show a gain of 0.008 in mean performance over the baseline, the results of Huang show a gain of 0.0047 in mean performance over the baseline, whereas the mean performance of our method is 0.0093 over the baseline. For the second category, as show in Table 3 and Fig 9(b), the results of Darabi show a gain of 0.06 in mean performance over the baseline, the results of Huang show a gain of 0.0062 in mean performance over the baseline, whereas the mean performance of our method is 0.0102 over the baseline.

**Table 2:** Image completion performance of the first three images in Fig 7 measured in SSIM.

Examples	Our	Darabi [20]	Barnes [12]	Huang [16]
(1)	0.9283	0.9209	0.9185	0.9226
(2)	0.9776	0.9718	0.9657	0.9731
(3)	0.9773	0.965	0.9713	0.9738
mean	0.9611	0.9526	0.9518	0.9565

**Table 3:** Image completion performance of the last three images in Fig 7 measured in SSIM.

Examples	Our	Darabi [20]	Barnes [12]	Huang [16]
(1)	0.7468	0.7424	0.7381	0.7415
(2)	0.8055	0.8016	0.7994	0.8032
(3)	0.7271	0.7227	0.7113	0.7258
mean	0.7598	0.7556	0.7496	0.7568

Regarding visual inspection, our approach produces better results than do the other methods in terms of image coherence and consistency. In addition, using the PSNR and SSIM comparison, we obtain a slightly higher PSNR value and SSIM value overall than do the other methods. Therefore, our approach performs better in terms of visual inspection, PSNR and SSIM.

## Conclusion

We proposed a new approach based on image depth for image completion. Our approach combines depth information and globally optimized texture synthesis to produce fewer artifacts and better consistency in image completion when compared to other state-of-the-art methods.

However, our approach does have several limitations. The main limitation concerns image depth and mismatched patches. To improve image completion performance, we want to examine some new directions in the future.

First, because we use a learning method to obtain the image depth, its accuracy is suspect, and an inaccurate image completion may result. To obtain an accurate image depth automatically, major technological advances in 3D scene understanding are required.

Second, our image completion method relies on a globally optimal solution by employing an expectation–maximization algorithm, which is easily influenced by random initial patches and tends to converge on local minima. In some unfavorable circumstances, some artifacts can be produced by this algorithm. Therefore, to obtain more consistent results, improving the optimization algorithms for a global optimal solution is required.
